# Does the application of whitening dentifrices during at-home bleaching affect the bond strength of resin composite to dentin?

**DOI:** 10.1186/s12903-022-02680-7

**Published:** 2022-12-26

**Authors:** Horieh Moosavi, Atefeh Nemati-Karimooy, Fatemeh Rezaei, Zahra Yavari, Farzaneh Ahrari

**Affiliations:** 1 Department of Restorative and Cosmetic Dentistry, School of Dentistry, Mashhad University of Medical Sciences, Mashhad, Iran; 2 I.M. Sechenova Medical Institute, Moscow, Russia; 3 Student Research Committee, Mashhad University of Medical Sciences, Mashhad, Iran; 4 Dental Research Center, School of Dentistry, Mashhad University of Medical Sciences, Vakilabad Blvd, Mashhad, Iran

**Keywords:** Bleaching, Dental bonding, Whitening dentifrice, Toothpaste, Bond strength, Ozone, Polymerization, Bleaching agents, Curing light, Composite resins

## Abstract

**Aim:**

This study aimed to evaluate the effect of using whitening dentifrices during at-home bleaching on the shear bond strength (SBS) of resin composite to dentin, and investigate whether the increased polymerization time would improve SBS.

**Methods:**

Ninety-six bovine incisors were divided into 4 groups of 24, according to the whitening treatment applied as follows: group 1, at-home bleaching + brushing with a regular dentifrice; group 2, at-home bleaching + brushing with a whitening dentifrice containing ozone; group 3, at-home bleaching + brushing with a commercial whitening dentifrice; and group 4 (control), no whitening/brushing treatment. Each group was divided into two subgroups (n = 12) to assess the effect of curing time (20 versus 80 s) on SBS. A self-etch adhesive was bonded to dentin, and after 2-day water storage, SBS was determined.

**Results:**

SBS was significantly affected by the whitening treatment (*P* = 0.03), but increasing the curing time had no significant effect on SBS (*P* = 0.137). Bond strength in group 1 was comparable to the control group (*P* > 0.05). The specimens in group 3 displayed significantly lower SBS than either group 4 or group 1 (*P* > 0.05). No significant difference was observed between the SBS of group 2 compared to any other group (*P* > 0.05). There was no significant association between the treatment group and failure type (*P* > 0.05).

**Conclusion:**

The bonding interface was not negatively influenced by the at-home bleaching procedure. However, using a commercial whitening dentifrice during at-home bleaching produced a significant detrimental effect on SBS. Extending the curing time would have no beneficial effect on adhesion to a whitened dental substrate.

## Introduction

As the desire to gain attractive smiles is growing, more patients request tooth whitening treatments. Personal dissatisfaction with tooth color has been reported in a relatively large percentage (56.2%) of the population [1]. Dental bleaching is a safe, minimally invasive, and popular technique to enhance the appearance of the teeth and smile esthetics. Bleaching can be performed through different techniques including in-office bleaching, at-home bleaching, and over-the-contour (OTC) whitening products. The in-office bleaching employs a highly concentrated hydrogen peroxide or carbamide peroxide gel to achieve a fast and effective result, whereas, for at-home bleaching, lower concentrations of peroxide are applied by the patients through self-worn trays [2–6]. There are different forms of over-the-contour products on the market such as tooth-whitening dentifrices, mouth rinses, varnishes, and strips; among them, dentifrices represent the most available, convenient, and commonly-used product. The ability of whitening dentifrices in both the elimination and prevention of stains has been demonstrated in several studies [2, 7, 8]. Therefore, some patients or practitioners may assume that the use of whitening dentifrices during professional bleaching would provide a synergistic effect on tooth color and enhance the esthetic outcome of therapy.

Despite its benefits, bleaching is associated with several harmful actions on tooth structure such as increased surface roughness, loss of mineral content, and degradation in mechanical strength of the bleached substrate [9–11]. These complications not only affect the tooth surface but also the underlying layers. Another side effect of tooth bleaching is the reduction in bond strength of restorations bonded immediately after the bleaching treatment [12, 13]. The reduction in adhesion strength has been mainly attributed to the alterations in morphological and mechanical properties of the bleached enamel/dentin, or to the presence of residual oxygen radicals within the dental tissues, which interfere with the polymerization process at the bonded interfaces [11–16].

Several strategies have been suggested to neutralize and reverse the effects of bleaching agents on bond strength such as delaying the restorative procedures up to 2 weeks after whitening [17–19] or treatment of the tooth surface with chemicals such as sodium ascorbate (a potent antioxidant), catalase, ethanol or water to dissociate residual peroxides and oxygen free radicals from the substrate [13, 20–23]. Another option to enhance the bond strength may be the increase in the polymerization time of adhesive systems. Caderano et al [13] measured the extent of polymerization of different adhesives on recently bleached dentin after curing for 20, 40, or 60 s. They reported a reduced extent of polymerization for all tested adhesives bonded to the whitened dentin substrate if the adhesive was bonded immediately after whitening and irradiated for 20 s. However, prolonged irradiation intervals (40 or 60 s) resulted in a significantly higher percentage of monomer conversion [13]. They suggested further studies to assess whether incomplete polymerization is responsible for the reduced bond strength in recently bleached teeth [13].

There are wide varieties of whitening dentifrices on the market. Some of them contain chemical or enzymatic ingredients that release free radicals to disintegrate the pigmented molecules; whereas others polish stains from the external surfaces of the teeth through abrasive particles. Recently, Ozone therapy has gained increasing attentiveness for the treatment of various conditions in medicine and dentistry. Ozone is a powerful oxidizing agent and demonstrates excellent sterilizing effects. It has been applied in the oral environment to promote wound healing, providing anti-caries and anti-gingivitis effects, and for disinfection purposes in endodontic and surgical procedures [24]. Ozone is not a stable element and should be applied through gas or liquid transmitters to be applied in regenerative medicine and dental practice. The ozonated olive oil is produced by infusing a high concentration of ozone in pure olive oil to benefit from both the moisturizing and smoothing properties of olive oil and the versatile properties of ozone. The effectiveness of ozone for lightening discolored teeth has been investigated in previous studies. Moosavi et al [25] demonstrated that the use of ozonated toothpaste leads to significant whitening of teeth from a clinical point of view. Al-Omiri et al [26] observed lighter shades on teeth bleached with both 38% hydrogen peroxide and ozone as compared to hydrogen peroxide alone. However, Zanjani et al [27] found that hydrogen peroxide had a more potent whitening effect than ozone.

Previous studies reported contrasting results about the bond strength of adhesive systems to dental substrates after the application of different whitening treatments [28–33]. However, little information is available regarding the bond strength of resin composite to teeth submitted to the combination of professional dental bleaching and brushing with whitening dentifrices. Therefore, the present study was conducted to assess the effects of a commercial whitening versus an ozonized whitening dentifrice applied during a 14-day at-home bleaching period on the shear bond strength between resin composite and dentin. The second aim of this study was to determine whether the increase in polymerization time is capable to improve the bond strength of resin composite to bleached dentin.

## Methods and materials

The sample of this experiment consisted of 96 freshly extracted and intact inferior bovine incisors obtained from sacrificed animals. The bovine teeth were used because of their large and flat surfaces and structural similarity to human teeth [34]. The teeth were cleaned from soft tissue remnants and then examined under a stereomicroscope to discard those with cracks, structural defects, or caries on the enamel surface. The selected teeth were kept in 0.1% thymol solution for 1 week, followed by immersion in 0.9% saline until the time of the experiment. The roots were cut by a diamond saw under water spray 1 mm below the cementoenamel junction. The crowns were mounted horizontally in self-curing acrylic resin so that the buccal surfaces were exposed for further experimental procedures.

The samples were numbered and randomly divided into 4 groups of 24 each, according to the whitening treatments applied, as follows:

- Group 1: at-home bleaching + brushing with an experimental, regular dentifrice
- Group 2: at-home bleaching + brushing with an experimental, whitening dentifrice containing ozonated olive oil at the concentration of 15 µg/ml
- Group 3: at-home bleaching + brushing with a commercial, whitening dentifrice (Aquafresh Intense clean whitening; Brentford, Middlesex, UK)
- Group 4 (control): no whitening/brushing treatment

Each group was then divided into two subgroups (n = 12) to assess the effect of curing time on dentin bond strength. In one subgroup, the composite was cured for 20 s, whereas in the other subgroup, the curing time was 80 s.

In groups 1–3, the dentin surfaces were covered by a 1 mm thickness of a 22% carbamide peroxide gel (Pola Night;‎ Southern Dental Industries, Australia) for 8 h a day [10]. The specimens were kept in an incubator at 37 °C and 100% humidity during the at-home bleaching treatment [10]. The samples were then washed with running tap water and exposed to brushing with the assigned dentifrice [35]. The dentifrice was applied in pea size over the surface, and the teeth were brushed in a circular motion using a power toothbrush (Oral-B professional care 3000; Procter and Gamble, Cincinnati, OH, USA) for 6 minutes to simulate daily tooth brushing. The bleaching and brushing treatments were performed for 14 days. The teeth were washed in tap water after brushing and stored in 0.9% saline solution at 37 °C between the whitening treatments. In group 4, the samples were stored in normal saline solution in the incubator without submission to any bleaching or brushing treatment.

Forty-eight hours after completing the 2-week bleaching and brushing period, the teeth were cut at the area between the middle and cervical thirds using a three-dimensional CNC cutting device. The cervical third of the tooth was selected for the experiment because of its lower enamel thickness. The enamel and superficial dentin were trimmed, then the dentin surface was ground flat by 600-, 1000- and 1200-grit silicon carbide abrasive papers under water cooling. The samples were irrigated for 2 minutes, then underwent the bonding process.

**Fig. 1:**
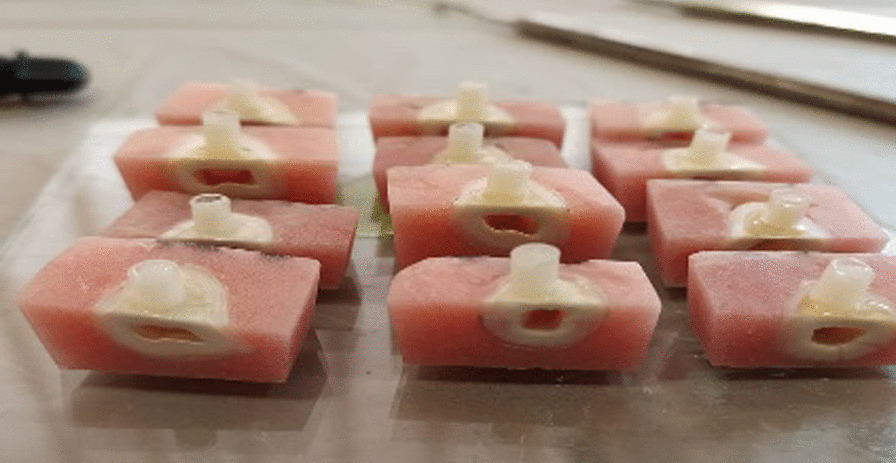
Insertion of resin composite into plastic molds for bonding to dentin

A self-etch adhesive system (Peak Universal Bond, Ultradent, South Jordan, UT, USA) was applied to the bonding area according to the manufacturer’s instructions. The adhesive was cured for 20 s using a light-emitting diode (LED) device (iLED Plus Curing Light; Woodpecker, China) at an intensity of up to 2300 mW/cm^2^. A plastic tube measuring 2.0 mm in diameter and 4.0 mm in height was used for the stabilization of composite resin on the dentin surface. The plastic tube was held perpendicular to the bonding area and the composite (Valux plus; 3M ESPE, St Paul, MN, USA) was inserted and condensed into it (Fig. 1). The excess was removed by a sharp explorer and the sample was light-cured for either 20 s (subgroup 1) or 80 s (subgroup 2). The bonded specimens were kept in distilled water for 48 hours at room temperature to complete the polymerization process. After that, the plastic tube was removed and the shear bond strength of the composite to dentin was assessed by a testing machine (Santam STM-20, Iran) using the crosshead speed of 1 mm/min. The maximum load at failure was recorded in newtons (N) and divided by the bonding area to express bond strength in MPa.

Following bond strength testing, the fractured surfaces were examined under a stereomicroscope at 40× magnification. The fracture mode was classified as adhesive (failure at the adhesive-tooth interface), cohesive (failure within the resin composite/dentin), or mixed (a combination of adhesive and cohesive failures).

Statistical analysis: The normal distribution of the data was confirmed by the Shapiro–Wilk test (*P* > 0.05). A two-way analysis of variance (ANOVA) was run to detect the effects of whitening treatment and curing time on the shear bond strength of resin composite to dentin, followed by the Tukey post hoc test for multiple comparisons. The difference in failure mode distribution was analyzed by Fisher’s exact test. The statistical analysis was performed using SPSS software (version 16.0 for Windows, SPSS Inc., Chicago, IL, USA), and the significance level was set at *P* < 0.05.

## Results

### Comparison of bond strength values among the groups

Table 1 presents the mean and standard deviation (SD) of shear bond strength values (MPa) in the sample. The two-way ANOVA displayed no significant interaction between the two variables treatment and curing time (*P* = 0.625). Shear bond strength to dentin was significantly affected by the whitening treatment (*P* = 0.03), however, increasing the curing time had no significant effect on SBS (*P* = 0.137).

The highest SBS was observed in group 4 (control). According to pairwise comparisons, the mean bond strength in the specimens submitted to at-home bleaching and brushing with the regular dentifrice (group 1) was not significantly different from the control untreated group (*P* > 0.05; Table 1). The dentin specimens in group 3 (at-home bleaching + brushing with the commercial whitening dentifrice) displayed significantly lower SBS than either group 4 or group 1 (*P* = 0.031 and *P* = 0.006, respectively). No significant difference was observed between the SBS of specimens submitted to at-home bleaching + brushing with the ozonized whitening dentifrice (group 2) compared to any other group (*P* > 0.05; Table 1).

**Table 1:** The mean and standard deviation (SD) of shear bond strength values (MPa) in the study groups at the different curing times

	Group 1		Group 2		Group 3		Group 4	
	At-home bleaching + brushing with a regular dentifrice		At-home bleaching + brushing with an ozonized whitening dentifrice		At-home bleaching + brushing with a commercial whitening dentifrice		Control (no whitening or brushing treatment)	
	Mean	SD	Mean	SD	Mean	SD	Mean	SD
20 s	8.26	3.41	7.72	4.06	6.17	3.11	9.87	4.11
80 s	7.98	2.11	6.52	3.66	6.03	2.56	7.57	1.69
Average*	8.12^a^	2.78	7.12^a,b^	3.83	6.10^b^	2.78	8.72^a^	3.29

*The groups that have been defined with different lowercase letters indicate a statistical difference at *P* < 0.05

### Comparison of failure mode distribution among the groups

**Fig. 2:**
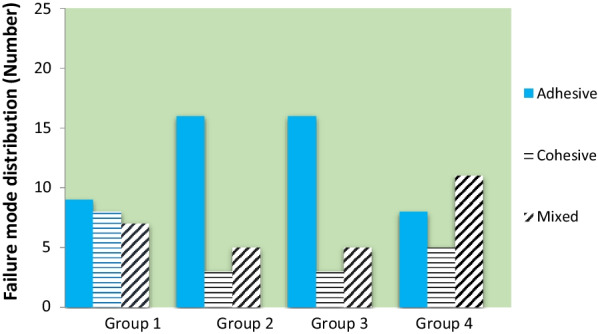
The overall distribution of fracture modes in the study groups

Table 2 presents the frequency of different failure types according to the bleaching treatment and curing time. In each group, a similar trend was found in the fracture mode distribution between the specimens subjected to 20 versus 80 s curing times (*P* > 0.05; Table 2). Overall, adhesive failure was the most frequent type in all groups, except group 4 (control), which showed a predominance of mixed fracture. The least frequently identified failure was the mixed mode in group 1, and the cohesive mode in the other groups. Figure 2 illustrates the overall distribution of fracture modes in the study groups. The statistical analysis revealed no significant association between the treatment group and the three types of failure, regardless of the curing time tested (*P* = 0.778).

**Table 2:** The frequency of failure modes according to the bleaching treatment and curing time

	Group 1			Group 2			Group 3			Group 4		
	At-home bleaching + brushing with a regular dentifrice			At-home bleaching + brushing with an ozonized whitening dentifrice			At-home bleaching + brushing with a commercial whitening dentifrice			Control (no whitening or brushing treatment)		
	Adhesive	Cohesive	Mixed	Adhesive	Cohesive	Mixed	Adhesive	Cohesive	Mixed	Adhesive	Cohesive	Mixed
20 s	4	5	3	7	3	2	8	2	2	3	2	7
80 s	5	3	4	9	0	3	8	1	3	5	3	4
*P* value	0.686			0.245			1.00			0.569		
Total	9	8	7	16	3	5	16	3	5	8	5	11

## Discussion

The present study investigated the effect of using whitening dentifrices during at-home bleaching on the shear bond strength of resin composite to dentin and assessed whether the increase in polymerization time can reverse the adverse effect of whitening on bond strength. Bovine incisors were used for the experiment, because of their availability, the extensive and flat tooth surface, and structural similarity to human teeth [34, 36, 37]. The total application time of brushing was 84 minutes, which is equal to an application of 2 min, three times a day for 2 weeks (the duration of at-home bleaching). Three types of dentifrices were used in this study. The first type was an experimental regular dentifrice. The ingredients of the experimental whitening dentifrice were similar to the regular dentifrice except for adding ozonized olive oil at the concentration of 15 µg/ml. The third dentifrice was a commercial whitening product (Aquafresh Intense clean whitening).

In the current study, the highest bond strength was observed in the control unbleached specimens (group 4). The samples submitted to at-home bleaching and brushing with the regular dentifrice (group 1) showed comparable bond strength to the control, unbleached dentin. Brushing with the ozonized whitening dentifrice during at-home bleaching (group 2) lead to a non-significant reduction in the bond strength of resin composite to dentin. The specimens in group 3 (at-home bleaching and brushing with the commercial whitening dentifrice) exhibited the least SBS value, which was significantly lower than both the control and the regular dentifrice counterparts. In all the study groups, bond strength at a curing time of 20 s was slightly greater than that of the 80 seconds curing time, but the difference was not statistically significant. The outcomes of this study indicate that the bonding interface is not negatively influenced by the at-home bleaching procedure if the restorations are bonded with a self-etch adhesive system 2 days after the completion of therapy. However, the use of a commercial whitening dentifrice during at-home bleaching can aggravate the adhesion and lead to a significant reduction in bond strength to recently bleached teeth. The findings of this study also imply that extending the polymerization time of adhesive systems would have no beneficial effect on adhesion to a whitened dentin surface. Therefore, clinicians should focus on other approaches to increase bond strength after tooth whitenings such as the extended waiting time before a restorative procedure or the application of an antioxidant on the bleached substrate [12, 13].

The reason for the reduced bond strength of bleached samples is under debate, but it is deemed that the residual peroxide and oxygen free radicals within the dental tissues are the main cause, acting by impeding appropriate composite polymerization [11, 13, 14]. The outcomes of this study, however, implied that the inhibition of polymerization is not a powerful mechanism to affect the SBS of resin composite to the whitened dental substrate, because the increased light irradiation period did not lead to enhanced adhesion strength. It appears that other factors are involved in jeopardizing the bond strength after the application of bleaching agents such as decreased mineral content and increased roughness and porosity of the enamel/dentin surface [11, 14]. Another factor that may contribute to the reduction in bond strength is the collapse or degradation of the dentin collagenous network as a result of dehydration or activation of metalloproteinase enzymes [12, 13]. The presence of residual oxygen inside the dental structure can also damage the bonding materials [38] or interfere with the formation of resin tags and the hybrid layer [21], and in this way, account for the decrease in bond strength values.

Concerning the fracture mode distribution, a similar trend was found between the specimens subjected to 20 versus 80 s curing times per study group. A predominance of adhesive fracture at the enamel-composite interface was observed in all groups except group 4 (control), whereas the least frequently identified fracture was the cohesive failure in most of the study groups. Previous studies also demonstrated a higher percentage of adhesive failure in the samples submitted to whitening treatments [11, 12, 39]. There was no significant difference in the distribution of failure mode among the study groups. In contrast, Torres et al [7] reported that the use of whitening dentifrices over the 12-month brushing period interfered with ARI scores, leading to a higher frequency of ARI score 3 (100% of the adhesive remained on the tooth surface) in whitened teeth.

There is limited research about the combined effects of at-home bleaching and brushing with a whitening dentifrice on the bond strength of resin composite to dentin. Therefore, a direct comparison of the results of this study with other investigations is not possible. The present findings are in agreement with some studies that reported no harmful effect on the bond strength of restorations bonded to teeth after at-home bleaching [30–33, 39, 40]. In contrast, several studies indicated lower adhesion strength in teeth recently exposed to professional bleaching procedures [18, 23, 41–43]. The investigations on the use of whitening dentifrices on bond strength also reported controversial results, with some studies reporting detrimental effects [7, 11, 14], whereas others showed comparable [9, 44] or even higher [45] bonding values as compared to regular dentifrices. These differences may be related to the various constituents of dentifrices or the discrepancies in the methodology applied among the studies. Whether the net effect of whitening dentifrices on dentin bond strength would be, the present findings indicate that the use of whitening dentifrices during the process of at-home bleaching results in reduced bond strength of restorations bonded to whitened teeth. When the ozonized whitening dentifrice is applied, the SBS reduction would be non-significant, but the use of a commercial whitening dentifrice would produce a significant detrimental effect on adhesion to bleached specimens. Although the reason for this difference between the whitening dentifrices is not well clear, it is possible that the ozonized dentifrice produces fewer oxygen radicals within the dentin structure and thus produces less harmful action on bond strength at the expense of lower whitening efficacy. The findings of this study imply that practitioners and patients should be warned about the harmful action of using over-the-contour whitening products during the process of professional bleaching on the adhesion strength of future restorations. A previous study also suggested that whitening dentifrices should be applied following instead of during in-office bleaching procedures to prevent color recession and better maintain the treatment results [2].

One of the limitations of this study was the use of a self-etch resin composite for assessing bond strength, whereas other adhesive systems may produce different results on bleached specimens. Furthermore, the effect of aging protocols was not considered in the present study. However, some studies reported that exposure to long-term water storage or thermocycling did not significantly impair the bond strength to enamel/dentin [12, 14, 46]. Another limitation of this study was that the specimens were kept in normal saline solution between the whitening treatments instead of artificial saliva and this may differ from the clinical conditions where the remineralizing potential of saliva may repair the effects of whitening agents on tooth structure. The brushing treatment also was somewhat different from the clinical situation, as no water or saliva was used to dilute the dentifrices. It is suggested that future studies assess the effect of various aging protocols as well as other adhesive systems on the bond strength of direct and indirect restorations submitted to cumulative whitening treatments.

## Conclusions

Under the conditions used in this experiment, the following conclusions can be drawn:

- The dentin specimens submitted to at-home bleaching and brushing with the regular dentifrice displayed comparable bonding values with the control, unbleached dentin. Therefore, the bonding interface is not negatively influenced by the at-home bleaching procedure.
- The use of a commercial whitening dentifrice during at-home bleaching resulted in the least SBS value on dental substrates, which was significantly lower than both the control and the regular dentifrice counterparts.
- Brushing with the ozonized whitening dentifrice during at-home bleaching was associated with a non-significant reduction in the bond strength of resin composite to dentin compared with the regular dentifrice.
- Extending the irradiation time of adhesive systems would have no beneficial effect on adhesion to a whitened dentin surface.
- There was no significant association between the treatment group and the type of failure, however, the bleached specimens revealed a higher frequency of adhesive failure at the bonding interface.

## Data Availability

The datasets used and/or analyzed during the current study are available from the corresponding author upon reasonable request.
